# Supplementary material for: “Dare to feel full”—A group treatment method for sustainable weight reduction in overweight and obese adults: A randomized controlled trial with 5-years follow-up
Source: PLoS One. 2024 May 9;19(5):e0303021. doi: 10.1371/journal.pone.0303021 (PMC11081318; doi:10.1371/journal.pone.0303021)
Supplement: S1 File — (DOCX) [file pone.0303021.s002.docx]

FORSKNINGSPLAN

**Våga vara mätt – gruppbehandling för viktnedgång**

Ansvariga forskare: Kjell-Åke Alle, docent, överläkare

Sara Holmberg, med dr, spec allmänmedicin och företagshälsovård

Lena Lendahls, med dr, barnmorska

Forskningshuvudman: FoU Kronoberg, Landstinget Kronoberg

**Vetenskaplig frågeställning**

Konceptet ”Våga vara mätt” är en pedagogisk metod med gruppbehandling som bygger på regelbundna måltider och mat som utgår från nordiska näringsrekommendationer. Metoden fokuserar på vanlig mat – ingen särskild diet, betydelsen av blodsockerreglering och hur det kan påverkas genom val av mat, ökad kunskap om näringsinnehåll i mat samt medvetenhet om eget beteende och beteendeförändring. Metoden har utarbetats av en enskild distriktssköterska och har visat bestående viktnedgång hos ett begränsat antal personer som provat metoden. Det är angeläget att vetenskapligt testa metoden innan eventuell implementering i rutinverksamhet inom hälso- och sjukvården.

Syftet med forskningsprojektet är att studera effekten av ”Våga vara mätt”- konceptet för viktnedgång med fem års uppföljning i jämförelse med nu rekommenderad kostrådgivning och sedvanlig vård vid övervikt/fetma.

Huvudfrågeställningen är om gruppbehandling enligt Våga vara mätt leder till bestående viktnedgång? Sekundära frågeställningar är om gruppbehandling enligt Våga vara mätt leder till förbättrad metabol profil och/eller ökad livskvalitet?

Hypotesen är att gruppbehandling enligt Våga vara mätt-konceptet ger bestående viktnedgång med 5 kg mer än vid kostrådgivning enligt aktuella riktlinjer vid övervikt/fetma. Sekundära hypoteser är att gruppbehandlingen leder till bättre metabol profil och/eller bättre livskvalitet.

**Områdesöversikt**

Fetma är en global epidemi och prevalensen av övervikt och fetma har dramatiskt ökat inom större delen av världen under den senaste 20-30-års perioden (1,2). Statistik från WHO visar att 1,5 miljarder vuxna var överviktiga 2008, varav en halv miljard feta (3). Samma utveckling har delvis skett i Sverige (4). Enligt färska siffror från SCB var 53% av männen överviktiga eller feta 2010, motsvarande siffra för kvinnorna 37%. I vardera gruppen klassades 15,5% respektive 14,4% som feta 2013.

Övervikt/fetma har i ett flertal epidemiologiska studier visat sig öka mortalitet och morbiditet för ett flertal sjukdomar och bidrar till kraftigt reducerad livskvalitet (5). I Framinghamstudien fann man att medellivslängden i genomsnitt förkortades 3 år vid övervikt samt ca 6-7 år vid fetma (6).

Samhällets kostnader för feta kan delas upp i direkta kostnader för behandling och omvårdnad och indirekta kostnader som konsekvens av sjukdom och behandling inkluderande frånvaro från arbetet och oförmåga att arbeta överhuvudtaget. Enligt socialstyrelsens beräkningar 1999 tros samhällsbelastningen för övervikt/fetma vara orsak till 2-4% av kostnaderna för sjukvården (7). Den beräknade återstående medellivslängden för Sveriges befolkning har ständigt ökat varför fetmaepidemin ännu ej påverkat befolkningens livslängd (8).

Sedan 50 talet har ett antal olika principer för viktminskning introducerats (9-12). Gemensamt för dem alla är att det saknas tillförlitliga långtidsdata, dvs hur många som efter 4-5 år lyckats att gå ner 5-10% av ursprungsvikten samt behålla den där (13,14). Ett flertal studier har visat att vikten kan reduceras signifikant på kort sikt (<2 år) nästan oberoende av vilket koncept som används (15,16). Fysisk aktivitet (FYSS-Fysisk aktivitet i sjukdomsprevention och sjukdomsbehandling) visar sig kunna minska viktuppgången efter lyckad viktnedgång, men långtidsstudier saknas (17). Mental styrketräning i form av KBT har använts i små grupper med kraftig övervikt med relativt goda korttidsresultat, dock inga långtidseffekter (18). På senare år har motiverande samtal blivit populärt, långtidsresultat på metodens effekt vid övervikt/fetma saknas (19).

Studier på längre sikt dvs 3-5-10 år har således mestadels varit nedslående och enbart en minoritet av de kvarvarande deltagarna kan anses ha en viktnedgång som är kliniskt signifikant dvs (5)-10% viktnedgång jämfört med ursprungsvikten. Huvudproblemet är därför att kunna bibehålla en signifikant viktnedgång över tid. Bariatrisk kirurgi har visat goda långtidsresultat över 20 år vad gäller viktnedgång och morbiditet men ej för sjukvårdskonsumtion (20). Operativa metoder är dock inte helt okomplicerade och kan bara användas för en minoritet av en fet befolkning.

Vi ämnar därför implementera en ny metod där vanlig husmanskost med beaktande av speciellt sockerfaran (21) som vi anser har stor betydelse för kostregleringen.

I korthet: modern mat sätter sockerbalansen ur spel som i sin tur påverkar hormonbalansen (könshormoner, stresshormoner, sömnreglering och hunger/mättnad balansen) och belöningssystemet. Detta ger störningar i fettomsättning (kulmage, blodfetter, LDL), fertilitet (PCOS), blodtryck och matintagsreglering. Man äter idag så man blir sugen och hungrig och inte så man blir mätt. En radikal sänkning av snabba kolhydrater leder till lättare hantering av matsuget och en återreglering av hormonstörningen. Därmed blir det möjligt att minska vikten långsiktigt. Vår metod använder en speciellt framtagen tallriksmodell och där målvikten utgör grunden för varje individs kaloriberäkning. I konceptet ingår att försökspersonerna kommer att stödjas intensivt under 6 månader med gruppmöten och individuellt framtagen information i likhet med beskriven svensk metod (22).

**Projektbeskrivning**

Studiedesignen är en randomiserad kontrollerad studie med fem års uppföljning där gruppbehandling enligt Våga vara mätt-konceptet jämförs med enskild kostrådgivning i enlighet med socialstyrelsens riktlinjer vid övervikt och fetma.

Grupp 1. Våga vara mätt – gruppbehandling under 6 månader (interventionsgrupp).

Grupp 2. Rekommenderad kostrådgivning enligt socialstyrelsens riktlinjer vid övervikt och fetma (kontrollgrupp).

Interventionsgruppen erbjuds 10 träffar i grupp om 6-8 deltagare under 6 månader. Ett individuellt samtal inkluderande provtagning hålls efter gruppträff 1 och 10. Vid gruppträff 5 ingår också ett kort individuellt samtal.

Kontrollgruppen erbjuds kostrådgivning enligt socialstyrelsens riktlinjer vid övervikt och fetma (inkluderande broschyrer) vid ett enskilt tillfälle inkluderande provtagning.

***Datainsamling***

Vid det första enskilda besöket (baseline) får deltagaren svara på en enkät med frågor om bakgrundsfaktorer, levnadsvanor, livskvalitet, hälsa och sjukdom. Provtagning genomförs av sjuksköterska inkluderande längd, vikt, bioimpedans, midje- och stuss mått, blodtryck, puls, venösa blodprover (blodstatus, blodfetter, ämnesomsättning, långtidsblodsocker och IGF-1). Blodproverna kommer att analyseras vid kliniskt kemiska laboratoriet vid Växjö Centrallasarett.

En uppföljningsenkät och provtagning upprepas på motsvarande sätt efter 6 månader och därefter årligen i 5 år på samma sätt för båda grupperna. Sammanlagt kommer sju undersökningar att görs av en sjuksköterska och deltagaren får ett testprotokoll för varje tillfälle.

Enkätsvar, mätningar och provsvar märks med löpnummer och införs till en forskningsdatabas.

En pilotstudie, innebärande en gruppbehandlingsomgång, kommer att göras innan rekrytering av forskningspersoner i syfte att säkerställa att konceptet fungerar praktiskt avseende gruppträffar, innehåll, enkäter, checklistor etc. samt för samordning/utbildning av medverkande gruppledare (2 sjuksköterskor plus reserver)

***Forskningspersoner***

Deltagarna till projektet kommer att rekryteras genom annonsering i dagstidningar i Kronoberg och Kalmar län. Efter kontakt med intresseanmälan görs telefonintervju av sjuksköterska för inklusion och information om projektet.

Inklusionskriterier: ålder 18-70 år, BMI 27-45, klarar svenska språket.

Exklusionskriterier: insulinbehandlad diabetes, grav psykisk sjukdom, grav lever eller njursjukdom, hjärtsvikt grad 3-4, annan allvarig allmänpåverkande sjukdom, multipel födoämnesallergi.

Avgränsningar avseende språk och allvarlig sjukdom görs för att projektet ska vara praktiskt genomförbart och inte innebära medicinska risker för deltagarna.

Den som vid telefonintervju uppfyller kriterierna och vill delta får ett informationsbrev hemsänt tillsammans med formulär för informerat skriftligt samtycke att återsända. Därefter lottas deltagarna till interventions- respektive kontrollgrupp.

***Statistiska överväganden och analys***

En powerberäkning har visat att vid alfa 0.05 och beta 0.2 (power 0.8) behövs 65 personer som randomiseras i vardera gruppen för att hitta en viktskillnad om minst 5 kg. Med hänsyn tagen till ett bortfall om ca 1/3 behöver 100 personer randomiseras till vardera gruppen.

Statistisk analys kommer att göras enligt intention-to-treat med huvudutfall viktnedgång. Ålder, kön, levnadsvanor, sjukdomar, socioekonomiska faktorer (fångas via enkäterna) bedöms vara väsentliga förväxlingsfaktorer att inkludera i analyserna.

Uppföljningsenkäten innehåller en öppen fråga om upplevelse/erfarenhet av deltagande i projektet. Dessa uppgifter kommer att analyseras med latent innehållsanalys.

**Betydelse**

Övervikt och fetma är ett växande folkhälsoproblem och leder till medicinska komplikationer, som för med sig minskad livskvalitet för individen och höga kostnader för hälso- och sjukvården. I dag drabbas unga överviktiga personer av sjukdomar som tidigare förknippades med hög ålder. Att utveckla och värdera icke-kirurgiska metoder för behandling/rådgivning vid övervikt och fetma med bestående resultat är mycket angeläget så väl för individ som samhälle.

**Preliminära resultat**

Några preliminära resultat finns inte. Gruppbehandlingsmetoden "Våga vara mätt" har använts för ett begränsat antal grupper såväl inom som utanför hälso- och sjukvården. Metoden har upplevts fungerat väl i praktiken men är inte prövad under kontrollerade former. Det har varit låg andel avhopp och många deltagare har uttryckt tillfredsställelse med gruppträffarna och sitt resultat.

**Etiska överväganden**

Deltagande i denna studie bedöms inte medföra några medicinska risker för deltagarna. Även kontrollgruppen erbjuds rådgivning och provtagning av erfarna rådgivare. Provtagningen kan medföra visst obehag men genomförs med gängse metod av erfaren personal. Gravt patologiska prover kommer att följas upp leg. läkare. Deltagarna kan förväntas få en hälsovinst i form av viktnedgång och bättre välbefinnande. Deltagandet är frivilligt och forskningspersonerna kan när som helst avbryta sitt deltagande utan att ange orsak till detta. Konfidentialitet garanteras och resultatet kommer att redovisas på gruppnivå vilket innebär att ingen enskild person kan identifieras.

**Referenser**

1. Prevalence and trends in obesity among US Adults, 1999-2008. Flegal KM, Carrolli MD, Ogden CL et al. JAMA 2010; 303(3): 235-241.
2. Increases in clinically severe obesity in the United States, 1986-2000. Sturm R. Arch Intern Med. 2003; 163:2146-2148.
3. International Obesity Task Force (IOTF); WHO rapport 2010.
4. Folkhälsan i Sverige. Årsrapport 2013. [www.Socialstyrelsen.se](http://www.Socialstyrelsen.se), mars 2013; Artikelnr: 2013-3-26.
5. Global status report on noncommunicable diseases 2010. Descrption of the global burden of NCDs, their risk factors and determinants. Edtors:World Health Organization. ISBN: 978-92-4-156422-9.
6. Obesity in adulthood and its consequences for life expectancy: A life-table analysis. Peeters A. et al. Ann Intern Med. 2003; 138:24-32.
7. Socialstyrelsen, Socialstyrelsens hemsida, 2008-02-15, <http://www.sos.se>.
8. Återstående medellisvlängd från åren 1751-2013. Kompleterad 2014-04-25. http://www.scb.se/sv_/Hitta-statistik/Statistik-efter-amne/Befolkning/Befolkningens-sammansattning/Befolkningsstatistik/25788/25795/Helarsstatistik---Riket/25830/
9. Low-fat dietary pattern and weight change over 7 years. Howard B, Manson J, Stefanick M et al. The women’s health initiative dietary modification trial.. JAMA 2006; 295, 39-49.
10. Medical obesity treatment: Long-term success in a primary care setting. Carney DM, Schultz SR, Carney SM. J Diabetes Sci Technol 2008; 1526.
11. Comparison of strategies for sustaining weight loss. Svetkey LP, Stevens VJ, Brantley PJ et al. The weight loss maintenance randomized controlled trial. JAMA 2008; 299 (10): 1139-1148.
12. Long-term weight-loss maintenance: a meta-analysis of US studies. Anderson JW, Konz EC, Frederich RC and Wood CL. Am J Clin Nutr 2001; 74: 579-84.
13. Medicare’s search for effective obesity treatments. Mann T,Tomiyama AJ, Westling E et al. Diets are not the answer. American Psychologist 2007; 62 (3): 220-233.
14. Dietary therapy for obesity: an emperor with no clothes. Mark, A. L. Hypertension 2008; 51(6): 1426-1434.
15. Rapport: Mat vid fetma (218/2013), ISBN:978-91-85413-59-1.
16. Weight loss with a low-carbohydrate, Mediterranean, o low-fat diet. Shai I, Schwarzfunchs D, Henkin Y et al. N Engl J Med 2008; 359: 229-41.
17. Statens folkhälsoinstitut/Yrkesföreningar för fysisk aktivitet, (2005), FYSS-Fysisk aktivitet i sjukdomsprevention och sjukdomsbehandling, Ödeshög, AB Danagårds Grafiska.
18. Cognitive-behavioural treatment for weight loss in primary care: a prospective study. Eichler K, Zoller M, Steurer J, Bachmann LM. Swiss Med Wkly 2007; 137: 489-495.
19. A randomized controlled trial of two weight-reducing short-term group treatment programs for obesity with an 18-month follow-up. Stahre L, Tärnell B, Håkansson C-E, Hällström T. Int J Behav Med 2007;14: 48-55.
20. Weight loss and health care use during 20 years following bariatric surgery. Neovius M, Narbro K, Keating C, Pelttonen M, Sjöholm K, Ågren G, Sjöström L, Carlsson L. JAMA 2012; 308(11):1132-41.
21. Cardiovascular disease resulting from a diet and lifestyle at odds with our paleolithic genome: How become a 21st-century hunter-gatherer. O’Keefe JH, Cordain L. Mayo Clinic Proc. 2004;79:101-108.
22. Hjärnkoll på vikten. 2014. David Ingvar, Gunilla Eldh. ISBN: 9789127129672.
