# Supplementary material for: “Dare to feel full”—A group treatment method for sustainable weight reduction in overweight and obese adults: A randomized controlled trial with 5-years follow-up
Source: PLoS One. 2024 May 9;19(5):e0303021. doi: 10.1371/journal.pone.0303021 (PMC11081318; doi:10.1371/journal.pone.0303021)
Supplement: S2 File — (DOCX) [file pone.0303021.s003.docx]

Translated document

**Trial Study Protocol – the RESEARCH PLAN attached to the Ethical Committee in 2014**

Dare to feel full – Group Treatment for Weight Loss

Principal Investigators:

Kjell-Åke Alle, Associate Professor, Chief Physician

Sara Holmberg, Ph.D., M.D., Specialist in General Medicine and Occupational Health

Lena Lendahls, Ph.D., Midwife

Research Principal: Research and Development Kronoberg, Kronoberg County Council

**Scientific Question**

The concept "Dare to feel full" is an educational method involving group treatment based on regular meals and food following Nordic nutritional recommendations. The method emphasizes regular food - no specific diet, the importance of blood sugar regulation, and how it can be influenced by food choices, increased knowledge of nutritional content in food, as well as awareness of one's own behavior and behavior change. The method was developed by an individual district nurse and has shown lasting weight loss in a limited number of people who have tried the method. It is essential to scientifically test the method before potential implementation in routine healthcare.

The aim of the research project is to study the effect of the "Dare to feel full" concept on weight loss with a five-year follow-up, comparing it with currently recommended dietary advice and standard care for overweight/obesity. The main research question is whether group treatment according to the Dare to feel full concept leads to lasting weight loss. Secondary questions include whether group treatment according to Dare to feel full leads to improved metabolic profile and/or increased quality of life.

The hypothesis is that group treatment according to the Dare to feel full concept results in lasting weight loss of 5 kg more than dietary advice following current guidelines for overweight/obesity. Secondary hypotheses are that the group treatment leads to a better metabolic profile and/or better quality of life.

**Literature Review**

Obesity is a global epidemic, and the prevalence of overweight and obesity has dramatically increased worldwide over the past 20-30 years. Statistics from the WHO show that 1.5 billion adults were overweight in 2008, of which half a billion were obese. The same trend has partly occurred in Sweden. According to recent figures from Statistics Sweden (SCB), 53% of men were overweight or obese in 2010, with the corresponding figure for women being 37%. In each group, 15.5% and 14.4%, respectively, were classified as obese in 2013.

Overweight/obesity has been shown in several epidemiological studies to increase mortality and morbidity for various diseases and contribute to a significantly reduced quality of life. Society's costs for obesity can be divided into direct costs for treatment and care and indirect costs as a consequence of illness and treatment, including absence from work and the inability to work at all.

The estimated remaining life expectancy for the Swedish population has constantly increased since the 1950s, which is why the obesity epidemic has not yet affected the population's life expectancy. Since the 1950s, various principles for weight loss have been introduced. Common to all of them is the lack of reliable long-term data, i.e., how many people have managed to lose 5-10% of their original weight and maintain it after 4-5 years.

Several studies have shown that weight can be significantly reduced in the short term (<2 years) almost independently of the concept used. Physical activity (FYSS-Physical activity in disease prevention and treatment) has been shown to reduce weight gain after successful weight loss, but long-term studies are lacking. Cognitive-behavioral therapy (CBT) in the form of mental strength training has been used in small groups with severe obesity with relatively good short-term results but no long-term effects.

In recent years, motivational interviewing has become popular, but long-term results on the method's effectiveness in overweight/obesity are lacking. Studies over the longer term, i.e., 3-5-10 years, have mostly been discouraging, with only a minority of the remaining participants having clinically significant weight loss (5-10%) compared to their original weight. The main problem is, therefore, to maintain significant weight loss over time.

Bariatric surgery has shown good long-term results over 20 years regarding weight loss and morbidity but not for healthcare consumption. However, operative methods are not entirely uncomplicated and can only be used for a minority of an obese population.

Therefore, we intend to investigate a new method with traditional home-cooked meals, taking current knowledge on risks with sugar into account, which are considered to have a significant impact on dietary regulation. In short: modern food disrupts the sugar balance, which in turn affects hormone balance (sex hormones, stress hormones, sleep regulation, and hunger/satiation balance) and the reward system. This leads to disturbances in fat metabolism (belly fat, blood lipids, LDL), fertility (PCOS), blood pressure, and food intake regulation. People today eat to become eager and hungry, not to become satiated. A radical reduction in fast carbohydrates leads to easier management of food cravings and a regulation of hormone disruption. Thus, it becomes possible to reduce weight in the long term. Our method uses a specially designed plate model, where the target weight forms the basis for each individual's calorie calculation. The concept includes that participants will be intensively supported for 6 months with group meetings and individually tailored information, similar to the described Swedish method.

**Project Description**

The study design is a randomized controlled study with a five-year follow-up, where group treatment according to the Dare to feel full concept is compared with individual dietary advice according to the National Board of Health and Welfare's guidelines for overweight and obesity.

Group 1: Dare to feel full – group treatment for 6 months (intervention group).

Group 2: Recommended dietary advice according to the National Board of Health and Welfare's guidelines for overweight and obesity (control group).

The intervention group is offered 10 group sessions with 6-8 participants over 6 months. An individual consultation, including sampling, is held after group session 1 and 10. Group session 5 also includes a short individual consultation. The control group is offered dietary advice according to the National Board of Health and Welfare's guidelines for overweight and obesity (including brochures) at a single occasion, including sampling.

**Data Collection**

At the first individual visit (baseline), participants answer a questionnaire about background factors, lifestyle, quality of life, health, and illness. Sampling is carried out by a nurse, including height, weight, bioimpedance, waist and hip measurements, blood pressure, pulse, venous blood samples (blood count, blood lipids, metabolism, long-term blood sugar, and IGF-1). The blood samples will be analyzed at the clinical chemical laboratory at Växjö Central Hospital. A follow-up questionnaire and sampling are repeated similarly after 6 months and then annually for 5 years in the same way for both groups. In total, seven examinations will be conducted by a nurse, and the participant will receive a test protocol for each occasion. Questionnaire responses, measurements, and test results are marked with a serial number and entered into a research database.

A pilot study, involving one group treatment session, will be conducted before recruiting research subjects to ensure that the concept works practically regarding group sessions, content, questionnaires, checklists, etc., and for coordination/training of participating group leaders (2 nurses plus reserves).

**Research persons**

Research participants in the project will be recruited through advertising in newspapers in Kronoberg and Kalmar counties. After contacting an expression of interest, a nurse conducts a telephone interview for inclusion and information about the project.

Inclusion criteria: age 18-70 years, BMI 27-45, proficiency in the Swedish language.

Exclusion criteria: insulin-treated diabetes, severe mental illness, severe liver or kidney disease, heart failure grade 3-4, other severe general affecting illness, multiple food allergies. Limitations regarding language and serious illness are made to ensure that the project is practically feasible and does not pose medical risks to the participants.

Those who meet the criteria during the telephone interview and want to participate will receive an information letter sent to their homes, along with a form for informed written consent to return. Subsequently, participants are randomized to the intervention or control group.

**Statistical Considerations and Analysis**

A power calculation has shown that with alpha 0.05 and beta 0.2 (power 0.8), 65 people need to be randomized in each group to find a weight difference of at least 5 kg. Considering a dropout rate of about 1/3, 100 people need to be randomized to each group.

Statistical analysis will be performed according to the intention-to-treat with the main outcome of weight loss. Age, gender, lifestyle, diseases, socioeconomic factors (captured through the questionnaires) are considered essential confounding factors to include in the analyses.

The follow-up questionnaire includes an open question about the experience of participating in the project. These data will be analyzed using latent content analysis.

**Significance**

Overweight and obesity are growing public health problems leading to medical complications, resulting in reduced quality of life for the individual and high costs for healthcare. Today, young overweight individuals suffer from diseases previously associated with old age. Developing and evaluating non-surgical methods for treatment/advice for overweight and obesity with lasting results is crucial for both the individual and society.

**Preliminary Results**

There are no preliminary results. The group treatment method Dare to feel full has been used for a limited number of groups both within and outside healthcare. The method has been perceived to work well in practice but has not been tested under controlled conditions. There has been a low dropout rate, and many participants have expressed satisfaction with the group sessions and their results.

**Ethical Considerations**

Participation in this study is not expected to pose any medical risks to the participants. The control group is also offered advice and sampling by experienced advisors. Sampling may cause some discomfort but is carried out with standard methods by experienced personnel. Grossly pathological test results will be followed up by a licensed physician. Participants can expect to gain health benefits in terms of weight loss and improved well-being. Participation is voluntary, and research subjects can withdraw from participation at any time without providing a reason for doing so. Confidentiality is guaranteed, and the results will be reported at the group level, meaning that no individual person can be identified.

**References**

1. Prevalence and trends in obesity among US Adults, 1999-2008. Flegal KM, Carrolli MD, Ogden CL et al. JAMA 2010; 303(3): 235-241.
2. Increases in clinically severe obesity in the United States, 1986-2000. Sturm R. Arch Intern Med. 2003; 163:2146-2148.
3. International Obesity Task Force (IOTF); WHO rapport 2010.
4. Folkhälsan i Sverige. Årsrapport 2013. [www.Socialstyrelsen.se](http://www.Socialstyrelsen.se), mars 2013; Artikelnr: 2013-3-26.
5. Global status report on noncommunicable diseases 2010. Descrption of the global burden of NCDs, their risk factors and determinants. Edtors:World Health Organization. ISBN: 978-92-4-156422-9.
6. Obesity in adulthood and its consequences for life expectancy: A life-table analysis. Peeters A. et al. Ann Intern Med. 2003; 138:24-32.
7. Socialstyrelsen, Socialstyrelsens hemsida, 2008-02-15, <http://www.sos.se>.
8. Återstående medellisvlängd från åren 1751-2013. Kompleterad 2014-04-25. http://www.scb.se/sv_/Hitta-statistik/Statistik-efter-amne/Befolkning/Befolkningens-sammansattning/Befolkningsstatistik/25788/25795/Helarsstatistik---Riket/25830/
9. Low-fat dietary pattern and weight change over 7 years. Howard B, Manson J, Stefanick M et al. The women’s health initiative dietary modification trial.. JAMA 2006; 295, 39-49.
10. Medical obesity treatment: Long-term success in a primary care setting. Carney DM, Schultz SR, Carney SM. J Diabetes Sci Technol 2008; 1526.
11. Comparison of strategies for sustaining weight loss. Svetkey LP, Stevens VJ, Brantley PJ et al. The weight loss maintenance randomized controlled trial. JAMA 2008; 299 (10): 1139-1148.
12. Long-term weight-loss maintenance: a meta-analysis of US studies. Anderson JW, Konz EC, Frederich RC and Wood CL. Am J Clin Nutr 2001; 74: 579-84.
13. Medicare’s search for effective obesity treatments. Mann T,Tomiyama AJ, Westling E et al. Diets are not the answer. American Psychologist 2007; 62 (3): 220-233.
14. Dietary therapy for obesity: an emperor with no clothes. Mark, A. L. Hypertension 2008; 51(6): 1426-1434.
15. Rapport: Mat vid fetma (218/2013), ISBN:978-91-85413-59-1.
16. Weight loss with a low-carbohydrate, Mediterranean, o low-fat diet. Shai I, Schwarzfunchs D, Henkin Y et al. N Engl J Med 2008; 359: 229-41.
17. Statens folkhälsoinstitut/Yrkesföreningar för fysisk aktivitet, (2005), FYSS-Fysisk aktivitet i sjukdomsprevention och sjukdomsbehandling, Ödeshög, AB Danagårds Grafiska.
18. Cognitive-behavioural treatment for weight loss in primary care: a prospective study. Eichler K, Zoller M, Steurer J, Bachmann LM. Swiss Med Wkly 2007; 137: 489-495.
19. A randomized controlled trial of two weight-reducing short-term group treatment programs for obesity with an 18-month follow-up. Stahre L, Tärnell B, Håkansson C-E, Hällström T. Int J Behav Med 2007;14: 48-55.
20. Weight loss and health care use during 20 years following bariatric surgery. Neovius M, Narbro K, Keating C, Pelttonen M, Sjöholm K, Ågren G, Sjöström L, Carlsson L. JAMA 2012; 308(11):1132-41.
21. Cardiovascular disease resulting from a diet and lifestyle at odds with our paleolithic genome: How become a 21st-century hunter-gatherer. O’Keefe JH, Cordain L. Mayo Clinic Proc. 2004;79:101-108.
22. Hjärnkoll på vikten. 2014. David Ingvar, Gunilla Eldh. ISBN: 9789127129672.
