# Supplementary material for: “Dare to feel full”—A group treatment method for sustainable weight reduction in overweight and obese adults: A randomized controlled trial with 5-years follow-up
Source: PLoS One. 2024 May 9;19(5):e0303021. doi: 10.1371/journal.pone.0303021 (PMC11081318; doi:10.1371/journal.pone.0303021)
Supplement: Attachment — Submitted filename: Response to reviewers.docx [file pone.0303021.s004.docx]

Response to reviewers

We thank the reviewers for reading critically and thereby contributing to improvement of our manuscript. Pleas find our response point-by-point below.

| Reviewer #1 |  |
| --- | --- |
| 1. Clarify what test was used for sample size calculation. Better use a method for repeated measures. | We have added the test name in the method section. |
| 2. The statistical analysis is not acceptable. For a longitudinal study with repeated measures, better consider longitudinal models such as mixed model and GLIMMIX models. | We consider our statistical analysis the most appropriate given the dataset, the size of the data material and the drop-out rate. The proposed methods would render estimated data results and we argue that such method would not alter the main results and it would not give more accuracy to the analysis. |
| 3. Specify primary endpoint (e.g. >5% reduction) and secondary endpoint. P value can be adjusted for secondary endpoint so power will not be reduced. | Primary outcome is already specified under statistical analysis in the method section. We have added all the additional secondary outcomes in the same place in the text.  All p-values were repeated analysis were performed have been adjusted with Bonferroni correction. |
|  |  |
| Reviewer #2 |  |
| The study is well written |  |
|  |  |
| 1. Define abbreviations used in tables/figures in the figure caption. | We have excluded abbreviations from table 1. In table 3, 4 and 7 we have added definition of abbreviations in footnotes. |
| 2. Mention the strengths and limitations of your study in a separate paragraph before the conclusions paragraph. | We have moved the paragraph about strengths and limitations of our study to just before the conclusion. We added a sub-heading “Strengths and limitations”. |
| 3. Add the clinical relevance of your study. | The clinical relevance of our findings is already presented in the last sentence of the first paragraph of the discussion and in the last five rows in the second paragraph of the discussion. We do not consider it relevant to elaborate further on this issue given the aim of the study. |
| 4. Add a new heading for conclusions paragraph. | We have added the suggested sub-heading. |
|  |  |
| Reviewer #3 |  |
| Specify the randomization process | We have clarified the writing on the randomization in the method section. |
| How is the eligibility criterion “being essentially healthy” is defined? | The definition was “not fulfilling exclusion criteria” and this has been added in a parenthesis. |
| Some tables (e.g. table 4, tables 7-8) should be moved to the supplementary appendix | We disagree with the proposal. We prefer to keep all the tables in the main manuscript. Regarding table 4 we consider these results important for the evaluation of the study due to the high drop-out rate. Table 7 and 8 reports some of the secondary outcomes and we consider the secondary outcomes equally relevant. |
| Could you provide in detail the classes of drugs taken “regularly” by 64% of enrolled subjects, according to data provided in table 2 (antihypertensives, hypolipidemic, antidiabetics?)? | We have provided brief information on type of medication in the first paragraph in the result section. |
| Despite the absence of an effect on HbA1c levels after 5 years of follow-up in both treatment arms, it would be interesting to know how many participants developed type 2 diabetes during study course. | We do not have access to complete diagnostic data or diagnoses within ordinary health care. Although of clinical interest data to answer this question was not part of our data collection. |
