# Supplementary material for: “Dare to feel full”—A group treatment method for sustainable weight reduction in overweight and obese adults: A randomized controlled trial with 5-years follow-up
Source: PLoS One. 2024 May 9;19(5):e0303021. doi: 10.1371/journal.pone.0303021 (PMC11081318; doi:10.1371/journal.pone.0303021)
Supplement: Attachment — Submitted filename: Response to reviewers.docx [file pone.0303021.s005.docx]

Response to reviewers

We are thankful to all the reviewers for valuable comments on our manuscript and we have now revised the manuscript further in accordance with the comments from Reviewer #1. We hope that with these amendments to our manuscript it will be suitable for publication. Please find our point by point response below.

| **Reviewer #1** |  |
| --- | --- |
| Tables 1-3 can be combined.  If a nonparametric method is used, better report median (IQR) instead of mean (SD). | Table 1 and 3 has been combined to a new table 1. Table 2 is left unchanged since it has another structure and a combination would be hard to read.  We have changed to Median and IQR. |
| Figure 2. bars are overlapping between two groups. | The bars in Figure 2 have been separated and the color changed to grey and black. |
| Table 4: this table can be omitted or moved to supplemental.  If a nonparametric method is used, better report median (IQR) instead of mean (SD).  Add percentages to treatment and sex.  MCV superscript can be removed.  Add “Abbreviations” to the footnote. | Previous Table 4 is now Table 3. We believe that this data should be kept in the manuscript for the best understanding of the data.  We have changed to Median and IQR.  We have added percentages to treatment and sex.  The MCV superscript has been corrected indicating statistical significance.  Abbreviations are included in the footnote. |
| Table 5: Why sample sizes are different for weight change, % change and reduction >=5% in control? E.g. 6m, 2 yr.. | Previous Table 5 is now Table 4. Sample sizes have been corrected. Thank for the attention, we discovered a mistake in our registry. |
| Table 5: my calculation for the p value of reduction >=2% is 0.01. Are other pvalues accurate? Weight % change and >=5% are secondary endpoint and p values may need to be adjusted. They can be presented in the table with other secondary endpoints. | We have only calculated for a reduction of >=5%, not 2%. Misunderstanding?  The p-value after Fisher exact test is 0.0063 but after correction with Bonferroni the p-value is 0.03 which is now stated. The other p-values have also been checked and are correct.  For best readability, we want to keep the secondary weight-related outcomes in Table 4 and have added this is the table heading. |
| Tables 6 and 7 can be combined. | Previous Table 6 and Table 7 have been combined and are now Table 5.  The p-values in the previous Table 8 which is now Table 6 have been corrected according to Bonferroni in accordance with the analyses with corrected numbers as for table 4. |
